# Function and Health in Adults with Dyskinetic Cerebral Palsy—A Follow-Up Study

**DOI:** 10.3390/jcm14144909

**Published:** 2025-07-10

**Authors:** Kate Himmelmann, Meta N. Eek

**Affiliations:** 1 Institute of Clinical Sciences, Sahlgrenska Academy, University of Gothenburg, 40530 Gothenburg, Sweden; 2 Institute of Neuroscience and Physiology, Sahlgrenska Academy, University of Gothenburg, 40530 Gothenburg, Sweden; meta.nystrom.eek@gu.se

**Keywords:** cerebral palsy, movement disorders, adult

## Abstract

**Background/Objectives**: Dyskinetic cerebral palsy (DCP) often implies severe motor impairment and risk of health problems. Our aim was to follow up a group of young adults with DCP that we previously examined as children, to describe health, function, and living conditions. **Methods**: Interviews regarding health issues, treatments, and living conditions, and quality of life (RAND-36) and fatigue questionnaires were completed. Gross and fine motor function, communication, and speech ability were classified, and weight, height, spasticity, and dystonia were assessed and compared to previous data. Joint range of motion (ROM) was compared to older adults with DCP. **Results**: Dystonia was present in all fifteen participants, and spasticity in all but two. A decrease was found mainly in those who received intrathecal baclofen (ITB). ROM limitations were most pronounced in shoulder flexion, abduction and inward rotation (while outward rotation was hypermobile), hip abduction, hamstrings, and knee extension. The majority had frequent contact with primary and specialist healthcare. Seven participants were underweight, eight had a gastrostomy, and seven had ITB. Upper gastrointestinal and respiratory problems were frequent. Orthopedic surgery for scoliosis was reported in five, and lower extremity in nine, while fractures were reported in six participants. RAND-36 revealed physical functioning, general health, and vitality as the greatest problem areas. Fatigue was significant in 64%. Eight participants lived with their parents. Participants at more functional levels completed tertiary education and lived independently. **Conclusions**: Most participants had severe impairment and many health issues, despite decreased dystonia and spasticity due to ITB. Sleep problems and pain were uncommon.

## 1. Introduction

The movement disorders of dyskinetic cerebral palsy (DCP) are defined according to the Surveillance of Cerebral Palsy in Europe (SCPE) [1] as “involuntary, uncontrolled, recurring, occasionally stereotyped movements. Primitive reflexes pattern predominate, muscle tone varies”. DCP comprises about 15% of the total group in population-based studies [2]. Children with DCP are mainly born at term and have frequently experienced adverse events at birth. The motor impairment is often severe, although a trend toward an improved motor function in recent birth-year cohorts has been reported from western Sweden [3]. Several treatments for dystonia in CP, such as intrathecal baclofen, deep brain stimulation, oral medication, and botulinum toxin, are at hand, as outlined in a review by Fehlings et al. [4]. Associated impairments are frequent. Intellectual impairment was found in 82%, epilepsy in 70%, and no understandable speech in 71% in a recent study, which also found ADHD, autism, or both diagnoses in 27% of children with DCP [5]. Lifespan research of individuals with DCP is limited, while the survival is known to be reduced compared to the general population [6].

The motor disability, accompanying impairments, growth, etiological factors, and neuroimaging were described in a group of children with DCP born in 1991–1998 in western Sweden [2]. The present study is a follow-up of individuals with DCP participating in this population-based study as children. It is part of a larger follow-up project in adults with CP (living as an adult with cerebral palsy in western Sweden).

Aim: To describe motor and accompanying impairments, dystonia, and signs of spasticity, anthropometry, and occurrence of pain compared to previous data in this group. Range of motion (ROM) limitations are compared to an older group with DCP from the larger adult follow-up project. Moreover, we aim to describe healthcare issues and treatments, occurrence of fatigue, and current life situation in terms of housing and occupation.

## 2. Materials and Methods

Participants were invited to a visit that included a physical examination, a semi-structured interview, and questionnaires, which covered past and present health concerns and feeding and living conditions. In addition, medical records were reviewed for information about medical history and treatments.

The following assessments were performed:

1. Gross motor function was classified with the Gross Motor Function Classification System (GMFCS) [7], manual ability with the Manual Ability Classification System (MACS) [8], and fine motor function with the Bimanual Fine Motor Function (BFMF) [9].
2. Dystonia, assessed at rest, was classified into two levels: very mild/mild or moderate/severe, corresponding to levels 1–2 and 3–4 of the Barry–Albright Dystonia Scale [10], and compared to previous assessment. Signs of spasticity in arms and legs were assessed with tendon reflexes and the Ashworth scale [11].
3. Range of motion was assessed and graded for severity: 1 = good mobility, 2 = light, 3 = moderate, and 4 = severe limitation, where grades 3 and 4 indicate limitations that could affect daily activities. The method has previously been described in detail [12].
4. Occurrence of pain was recorded from interviews, pain charts, and the RAND-36 dimension bodily pain (BP) [13], and also noted during the physical assessment.
5. Communication was classified with the Communication Function Classification System (CFCS), where levels I–II describe the ability to send and receive messages with an unknown partner [14]. Speech was classified with the Viking Speech Scale (VSS), describing the effect of motor impairment on speech [15].
6. Data on weight (kg), height (cm), waist circumference, and blood pressure were collected at the visit. BMI was calculated and compared to reference data for adults according to the WHO (underweight < 18.5, normal weight 18.5–24.9, overweight 25–29.9, and obesity > 30). Regarding information about eating and drinking, the Eating and Drinking Ability Classification System (EDACS) was considered [16].
7. Mental and physical health-related quality of life were assessed using the RAND-36 [13]. The questionnaire consists of 36 questions scored on a scale from 0 to 100, with 100 representing the highest level of functioning possible. Aggregate scores are compiled as a percentage of the total points possible, grouped in eight dimensions: physical functioning (PF), role limitations due to physical health (RP), bodily pain (BP), general health (GH), vitality (VT), social functioning (SF), role limitations due to emotional problems (RE), and mental health (MH).
8. Fatigue was evaluated with the Fatigue Severity Scale (FSS) [17]. The questionnaire consists of 9 items, and each item consists of statements that are scored on a 7-point scale ranging from 1 (“strongly disagree”) to 7 (“strongly agree”). The mean score of the items is used as the FSS score, where a high score indicates more fatigue, and with a cut-off of 4.0 for clinically significant fatigue.
9. Data on health problems, treatment, and living conditions were gathered from interviews and medical records.

Measurements in points 1–6 above were all assessed at both first examination and follow-up, except for ROM, where there were no data at first examination. Data for ROM were instead compared to that of older adults with DCP, previously published from this project [12], to get a comparison of severity of ROM limitations. Data from points 7–9 were only recorded at the follow-up.

To facilitate self-report of the questionnaires and interviews, adaptations were made using the talking mats methodology [18]. Five participants answered the questionnaires themselves (two of whom used talking mats), five with the support of an assistant or parent (two using a talking mat in the process), while a proxy completed the questionnaires in five cases.

### 2.1. Participants

Fifteen young adults participated. Background of CP was cerebral maldevelopment in two, perinatal hypoxic-ischemic encephalopathy in eleven, and kernicterus in one, while the background of CP was unknown in one participant.

### 2.2. Non-Participants

Of the 43 participants living in the county of Västra Götaland in the original study, two had moved out of the area.

Seven (three male) had died at a mean age of 17.8 years (range 11–30). They had severe motor and intellectual impairments and epilepsy, and dystonia was considered moderate to severe in four children at the initial assessment. The mean weight was low (SD −3.2). Five had a gastrostomy. Three of the seven children had been treated with an intrathecal baclofen pump (ITB) and five had received botulinum toxin injections.

Of the eligible non-participants (*n* = 19), we were not able to reach 15, and 4 declined to participate. At the first assessment, 14 were classified at GMFCS levels IV–V (8 at level V), with median MACS and BFMF at level IV. The dystonia was considered moderate to severe in 2 participants, spasticity signs in the legs were found in 16, and in the arms in 13. SD weight was −1.47, and mean BMI was 15.86. Six had a gastrostomy, and pain was reported in four. There was no statistically significant difference regarding the GMFCS level compared to the participants in the present study (*p* = 0.666) or speech ability (*p* = 0.436).

### 2.3. Older Comparison Group

For comparison of ROM, 29 older adults with DCP were examined with the same methods as part of the larger follow-up project in adults with CP. The older group consisted of 13 women and 16 men aged 38–57 years old (median 47), at the following GMFCS levels: I = 3, II = 4, III = 1, IV= 9, and V = 12. There was no statistically significant difference regarding the GMFCS level compared to the younger group (*p* = 0.189).

### 2.4. Statistical Analysis

Data consisted of nominal data, and ordinal scales and non-parametric tests were used for statistical analysis. The Chi2 test was used for comparison between groups, and the Sign test for comparison of changes within groups. A *p*-value less than 0.05 was considered statistically significant. Statistical testing was performed using SPSS version 29.0.2.0 (IBM Corp., Armonk, NY, USA).

### 2.5. Ethics

The study was conducted in accordance with the Declaration of Helsinki and approved by the Regional Ethics Review Board in Gothenburg (16 January 2014; No. 777-13) and the Swedish Ethical Review Authority (5 November 2019; No. 2019-05518).

The study was reported according to guidelines for reporting observational studies: “The Strengthening the Reporting of Observational Studies in Epidemiology” (STROBE) Statement, from the Equator network.

## 3. Results

Fifteen young adults (four women and eleven men) were examined. The first examination was performed at 5–11 years of age (median 9 years), and the second at 18–25 (median 23).

### 3.1. Motor Function

Most of the participants were found at the least functional level (level V) in all classifications, see Table 1. Improvements at follow-up were seen in three cases in MACS and one case in GMFCS, and deterioration in one case in GMFCS.

### 3.2. Dystonia and Spasticity

Dystonia/dyskinesia was present in all participants, noted as mild in eight participants and as moderate–severe in seven at first examination. Presence of dystonia/dyskinesia was scored as better (four), no change (ten), and worse (one) at follow-up, a difference that was not statistically significant. The four participants with less dystonia had all received intrathecal baclofen.

Signs of spasticity, tested with tendon reflexes and the Ashworth scale, were present in all but 2 participants (arms in 11 at first exam and 12 at follow-up, and 13 in the legs on both occasions). Less exaggerated reflexes were seen in five participants at follow-up, of whom four had received intrathecal baclofen.

Primitive reflexes were noted in 14 participants at first examination and in 9 at the follow-up, while they were not commented on in 5.

### 3.3. Range of Motion

**Figure 1:**
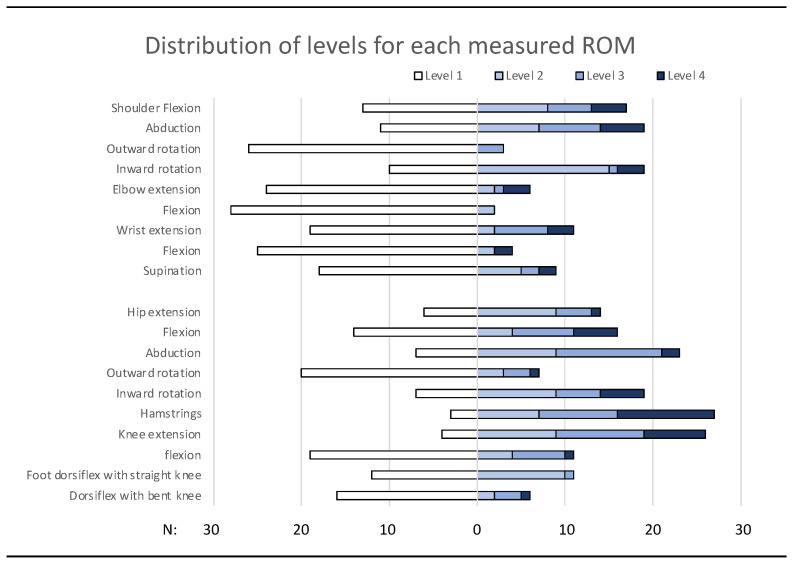
ROM in the upper and lower extremities, classified into four levels based on the severity of limitations: 1 = good ROM, 2 = mild limitation, 3 = moderate limitation, and 4 = severe limitation. Level 1 = good ROM is displayed to the left and the limitation levels 2–4 to the right. All 15 participants were measured on both the right and left sides, for a total of 30 measurements.

Range of motion limitations were common, and more pronounced in the lower extremities, see Figure 1 and Table 2. Regarding the upper extremities, limitations were more pronounced in the shoulder joint, with values potentially affecting functions (grades 3–4) for shoulder flexion (30% of the participants) and shoulder abduction (40%). It is notable that many were even hypermobile in outward rotation, with 87% having no limitation (grade 1). In contrast, inward rotation showed the reverse situation, with 63% having limitations (grades 2–4).

In the lower extremities, limitations affecting function were most common in hip flexion (40%), hip abduction (47%), hamstring muscles (67%), and knee extension (57%).

Results were compared with a group of older individuals with DCP examined with the same methods, showing a trend toward deterioration in the older group, see Table 2, mostly seen as a lower proportion with no limitation in the older group. Differences between the younger and the older group were statistically significant for outward rotation in the shoulder, hip abduction, and outward rotation in the hip.

### 3.4. Pain

Pain in different parts of the body was reported in seven participants at first examination, and in seven at follow-up (47%), however, not by the same individuals. Pain was graded as severe in one participant, and two reported pain to have lasted over three months.

### 3.5. Communication

Eight participants were classified at CFCS level V, three at level IV, and one at level III, while three were able to communicate with unfamiliar partners (CFCS I–II). The VSS revealed that ten out of eleven young adults, described as having anarthria at the first assessment, still had no intelligible speech, while one had gained some speech function, although severely affected by the motor impairment (VSS III). Augmentative and alternative communication was used by 12 participants (80%), ranging from high to low technology in different combinations. Symbols and pictures, together with body language, were used by eleven participants. High-technology resources, such as computer eye-tracking and other digital software, were reported to be used by five participants, compared to none in the first study.

### 3.6. Growth and Nutrition

Anthropometric measures are presented in Table 3. Regarding body mass index (BMI), seven participants were in the range for normative values, one was borderline overweight (BMI 25), while seven were considered underweight, despite that three of the latter had a gastrostomy. Based on BMI, this indicated a deterioration, although not significant, compared to our first assessment. Waist circumference showed low values (median 72, range 63–90).

Of the seven orally fed participants, five had problems corresponding to EDACS levels III–IV, and all needed assistance. Two were considered totally dependent. Two participants were at EDACS levels I–II, one of whom needed some assistance, while one was independent.

### 3.7. Health-Related Quality of Life

**Figure 2:**
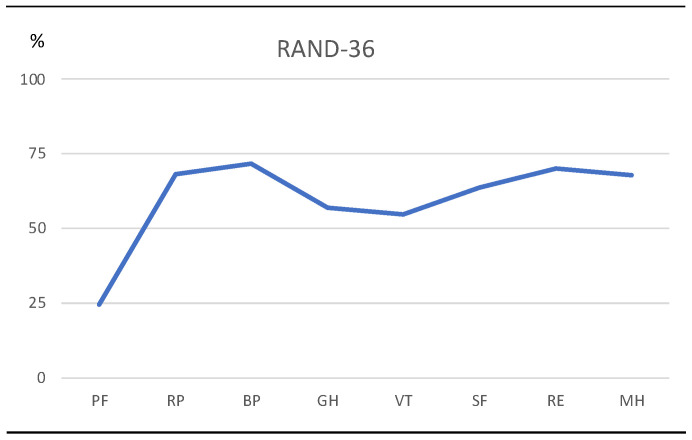
RAND-36 dimensions: physical functioning (PF), role limitations due to physical health (RP), bodily pain (BP), general health (GH), vitality (VT), social functioning (SF), role limitations due to emotional problems (RE), and mental health (MH). Here, 100% represents the highest level of functioning possible.

Information about health and functioning using the RAND-36 was available in eleven participants, but for the dimension physical functioning (PF), it was only registered for three participants. Results are presented in Figure 2. The greatest problem was physical functioning (PF), with a score of 24, followed by general health (GH) at 56.8 and vitality (VT) at 54.7, while the least problems were role limitation due to physical health (RP), bodily pain (BP), role limitation due to emotional problems (RE), and mental health (MH), all with scores around 70.

### 3.8. Fatigue

Nine participants completed all items of the FSS, while five participants left out one to three of the nine items. Dividing the score by the number of items reported gave a median of 4.3 (range 1.1–5.8). Nine participants (64%) scored over the cut-off of 4.0, indicating clinically significant fatigue.

### 3.9. Health Conditions and Treatments

All but one participant had frequent contacts with healthcare, both primary healthcare and adult multi-disciplinary habilitation services. Specialist services included respiratory medicine, spasticity clinic, and orthopedic and nutritionist services.

Seven participants (47%) were treated with intrathecal baclofen (initiated at age 10–17 years). Ongoing treatment with oral medication against dystonia and spasticity was reported in three cases and botulinum toxin treatment in four cases.

Eight participants (53%) had a gastrostomy, while five had oral feeding of mixed or otherwise prepared consistency. Only two did not need specially prepared food. Upper gastrointestinal (GI) disorder, such as reflux and esophagitis, was treated in six participants (40%), and lower GI problems were mainly related to obstipation.

Respiratory disorders, such as problems with phlegm and pneumonia, often recurrent, were reported in six participants (40%). Some of the recurrent pneumonias were associated with aspiration. Treatments ranged from inhalations and positive expiratory pressure masks to cough assist devices and ventilators, and continuous oxygen treatment in one case. One participant had received a tracheostomy.

Five participants (33%) had undergone surgery for scoliosis, and nine (60%) had lower extremity surgery (mainly hip and Achilles tendon surgery). Six (40%) had sustained fractures in upper or lower extremities, some of them repeatedly. These were reported to be spontaneous or occurring after minor trauma. Three had experienced pressure ulcers.

Fourteen participants (93%) reported to be sleeping well, while one reported disturbed sleep. Psychiatric and/or neuropsychiatric conditions (autism, anxiety, and depression) were reported in four cases. Nine (60%) took anti-epileptic medication.

### 3.10. Living Conditions and Social Outcome

None of the participants were married or had children. Eight (53%) lived with their parents, two lived in a group home, three lived alone with assistants, one lived independently with assistance on call, and one lived independently. Need for assistance in daily life, not only by staff but also by relatives, was reported in the majority of cases (87%).

Seven participants (47%) were students, two of whom were in tertiary education, while five followed an adapted school curriculum. Four had daily activities organized for persons with functional impairments, and two had part-time employment, subsidized or sheltered. Thirteen participants (87%) had activity or sickness compensation and two received student aid. Additionally, three received insurance compensation. In society, ten (67%) were transported in a wheelchair, four drove an electric or manual wheelchair, while one did not need a wheelchair. Thirteen used the mobility service for persons with functional impairments.

## 4. Discussion

DCP is often a severe motor condition. The participants in this follow-up were children at the time of the first study. The majority had a severe motor impairment but there were also milder cases in this group.

There was a decrease in spasticity, as well as dystonia. This was mainly found in individuals who received ITB. These individuals, who were at low functional levels, had an improved sitting function and less pain, spasticity, and dystonia compared to data from childhood and follow-up after ITB [2,19,20]. However, this did not hinder the occurrence of health issues in this group.

Previous research on ROM has often focused on the legs, showing increasing limitations with age, but with samples consisting mainly of spastic CP at GMFCS level I, making it difficult to compare to our data [21,22]. A study on older adults with CP presented data on both upper (nine muscle groups) and lower extremities (ten muscle groups), where moderate to severe limitations were present in 98% of the participants. Upper extremity limitations were more common in DCP, compared to the other subgroups [12]. In the present study of young adults with DCP, ROM limitations were already evident, and with a severity that could affect many functional abilities and have a large impact on daily life, such as dressing, sitting, mobility, and with a risk of pain and deterioration later in life. The limitations in the upper extremities in DCP, in combination with involuntary movements and lack of speech, can be especially harmful for the ability to communicate and use devices for augmentative and alternative communication. The comparison between our study group and older individuals with DCP showed a trend toward more limitations in the older group.

Communication and speech were classified with the CFCS [14] and the VSS [15] at follow-up. These classifications were not developed at the first study. Speech ability was then described as presence of anarthria and dysarthria, and the type of communication aids was reported. Communication function, known to be low in most of the children, had not changed at follow-up, but the communication aids were more advanced in the follow-up, probably due to increased technical development of aids, but also increased attention to the area of communication [23].

Median weight was low in the group, but also median height. In a recent study, adolescents with CP and GMFCS I–III reached similar heights as their typically developing peers, while those at GMFCS IV–V, much like the majority of the participants in the present study, did not [24]. Stunting makes BMI results a less reliable measure, as the undernutrition may be underestimated. Inadequate nutrition and increased energy expenditure due to dystonia and spasticity may affect growth, and in our study almost half were underweight. Of the seven orally fed participants, five had significant problems, corresponding to EDACS [14,16] levels III–IV, and needed various amounts of assistance.

Respiratory problems were common in our study, and they were sometimes linked with gastrointestinal problems. Increased muscle tone, muscular weakness, and scoliosis, combined with gastrointestinal reflux and poor airway clearance, predispose for infections and decreased respiratory function. As previously described, this is a frequent cause of hospital admissions [25]. Another problem of special concern was the occurrence of fractures. A recent systematic review found that occurrence of osteoporosis and fractures was high in adults with CP and severe motor impairment, and that the risk factors for poor bone health were low mobility, nutritional deficiencies, and anticonvulsant treatment [26]. Many participants in the present study had these risk factors.

Health-related quality of life (HRQoL) was assessed using the RAND-36. Compared to Swedish reference data for ages 20–29 years, all values were below the reference data, indicating poor HRQoL. Notable is that for vitality, scores were similar, and low, in both the study participants and the reference data [27]. Comparing to an Australian sample, a similar profile as in the present study emerged, except for vitality and social functioning, where the Australian study scored higher [28]. However, this was not directly comparable, as the latter study involved a mixed group and ages up to 66 years. A study on factors impacting the health and well-being of young adults with CP suggested that emotional support plays a significant role in the health status and satisfaction with life [29].

The occurrence of fatigue, as reported with the FSS, indicated clinically significant fatigue in two-thirds of the group. Presence of fatigue was also seen in the RAND-36 dimension vitality. The FSS score was similar to a previous study in adults with CP in ages 24–76 years [30] but higher than in a group in ages 20–22 years [31]. However, the populations in both these reports consisted mostly of spastic cerebral palsy at GMFCS levels I–IV.

Pain is often described as a problem in cerebral palsy populations [32]. In the present study, pain was reported in 47% of participants. This is less than in a group of older adults with DCP published from this project, with pain reported in 66% [33], and in a study from the Swedish Cerebral Palsy Follow-up Program (CPUP), where two-thirds of adults with DCP experienced pain [34]. The results of RAND-36, dimension bodily pain, confirmed that pain was not the dominant problem affecting function in the group in the present study.

Although transition to other housing arrangements had taken place in some cases, more than half of the participants, all with a severe impairment, still lived with their parents. In addition to receiving social support of different kinds, such as assistants [35], they also relied on the support of their parents in daily life. This is in line with the findings in a Swedish study of mixed CP types from the national quality register CPUP, where living with parents was associated with a severe motor impairment, as well as a younger age [36].

### Limitations

The study group was small, which affects the generalizability of the results. Seven of forty-three had died in the original population-based group, which is a high percentage. We have previously shown a reduced survival in DCP in the same population. Moreover, children and adults with CP and severe impairments in general have a reduced survival [6]. Also, we were not able to include 19 of the original, and eligible, participants. However, there was no significant difference in gross motor function or speech ability between the non-participants and the participants of the follow-up.

The research on DCP is still limited. Therefore, it is difficult to make direct comparisons with previous research, as many studies comprise of mixed subtypes, and mostly focus on spastic CP, which is the most common type of CP. However, a strength of the present study is that it is one of few studies that concern only DCP, and the changes from childhood to young adulthood in a well-known population. Therefore, the study provided useful information for planning of healthcare and prevention of secondary complications.

## 5. Conclusions

Our results showed that young adults with severe DCP have many healthcare problems. Despite this, the majority were sleeping well, and the occurrence of pain was low. Problems with dystonia, spasticity, and orthopedic issues were being addressed by healthcare in many cases. However, underweight, respiratory problems, contractures, and fractures were still common, and transition to adulthood was affected. Participants with a milder impairment were living independently and pursued tertiary education. As recent studies point toward a less severe clinical picture in DCP, and the management and treatment of DCP improve, a less severe outcome and improved opportunities for the individual may be more common.

## Figures and Tables

**Table 1:** Classification of participants for motor abilities at first examination (1) and follow-up (2).

	GMFCS 1	GMFCS 2	MACS 1	MACS 2	BFMF 1	BFMF 2
N = Level I	0	1	0	1	0	0
II	1	0	1	2	1	2
III	2	2	2	0	3	0
IV	3	2	4	3	3	4
V	9	10	8	9	8	8

Gross Motor Function Classification System (GMFCS), Manual Ability Classification System (MACS), and Bimanual Fine Motor Function (BFMF).

**Table 2:** ROM in the upper and lower extremities, classified into four levels based on the severity of limitations, presented in percent at each level, and comparison of the younger and older groups.

	Level 1 Good		Level 2 Mild		Level 3 Moderate		Level 4 Severe		*p*-Value
	Young	Old	Young	Old	Young	Old	Young	Old	
*Shoulder*	%	%	%	%	%	%	%	%	
Flexion	43	29	27	45	17	12	13	14	*n.s.*
Abduction	37	21	23	36	23	28	17	14	*n.s.*
Outward rotation	87	62	0	16	10	12	0	7	*0.037*
Inward rotation	33	34	50	36	3	19	10	5	*n.s.*
*Elbow*									
Extension	80	67	7	9	3	17	10	7	*n.s.*
Flexion	93	78	7	17	0	3	0	0	*n.s.*
*Wrist*									
Extension	63	57	7	19	20	10	10	10	*n.s.*
Flexion	83	79	7	9	0	7	7	2	*n.s.*
Supination	60	62	17	19	7	9	7	3	*n.s.*
*Hip*									
Flexion	47	28	13	29	23	22	17	19	*n.s.*
Abduction	23	3	30	26	40	53	7	12	*0.031*
Outward rotation	67	22	10	34	10	14	3	3	*0.003*
Inward rotation	23	14	30	19	17	21	17	21	*n.s.*
*Knee*									
Hamstrings	10	9	23	19	30	28	37	24	*n.s.*
Extension	13	26	30	22	33	31	23	19	*n.s.*
Flexion	63	60	13	14	20	17	3	5	*n.s.*
*Ankle*									
Dorsiflex bent knee	53	48	7	36	10	7	3	2	*n.s.*

n.s. = No statistically significant difference.

**Table 3:** Age and anthropometric data for first examination (1) and follow-up (2).

	Age 1	Age 2	Height 1 *	Height 2	Weight 1	Weight 2	BMI 1 *	BMI 2
Median	9	23	122.5	161	23.3	48.0	15.4	18.6
Max.	11.9	25	142	172	44.0	61.6	22	25
Min.	5.0	18	96	145	13.8	35.3	11.5	15.5
IQR	2.9	3.0	14.8	9.0	10.3	10.8	3.9	4.3

* Height was missing in one participant.

## Data Availability

The datasets presented in this article are not readily available because of privacy and ethical restrictions.

## Abbreviations

The following abbreviations are used in this manuscript:

DCP	Dyskinetic cerebral palsy
ROM	Range of motion
ITB	Intrathecal baclofen
SCPE	Surveillance of Cerebral Palsy in Europe
GMFCS	Gross Motor Function Classification System
MACS	Manual Ability Classification System
BFMF	Bimanual Fine Motor Function
CFCS	Communication Function Classification System
VSS	Viking Speech Scale
EDACS	The Eating and Drinking Ability Classification System
FSS	Fatigue Severity Scale
BMI	Body mass index
HRQoL	Health-related quality of life
CPUP	The Swedish Cerebral Palsy Follow-up Program
